# DSCW-YOLO: Vehicle Detection from Low-Altitude UAV Perspective via Coordinate Awareness and Collaborative Module Optimization

**DOI:** 10.3390/s25113413

**Published:** 2025-05-28

**Authors:** Qingqi Zhang, Hao Wang, Xinbo Wang, Jiapeng Shang, Xiaoli Wang, Jie Li, Yan Wang

**Affiliations:** Electronic Information Engineering College, Changchun University, Changchun 130022, China; zhangqq@ccu.edu.cn (Q.Z.); wangxb@ccu.edu.cn (X.W.); 230402184@mails.ccu.edu.cn (J.S.); wangxl@ccu.edu.cn (X.W.); lij69@ccu.edu.cn (J.L.); wangy8512@ccu.edu.cn (Y.W.)

**Keywords:** deep learning, UAV, object detection, YOLO

## Abstract

This paper proposes an optimized algorithm based on YOLOv11s to address the problem of insufficient detection accuracy of vehicle targets from a drone perspective due to certain scenes involving complex backgrounds, dense vehicle targets, and/or large variations in vehicle target scales due to oblique imaging. The proposed algorithm enhances the model’s local feature extraction capability through a module collaboration optimization strategy, integrates coordinate convolution to strengthen spatial perception, and introduces a small object detection head to address target size variations caused by altitude changes. Additionally, we construct a dedicated dataset for urban vehicle detection that is characterized by high-resolution images, a large sample size, and low training resource requirements. Experimental results show that the proposed algorithm achieves gains of 1.9% in precision, 6.0% in recall, 4.2% in mAP@0.5, and 3.3% in mAP@0.5:0.95 compared to the baseline network. The improved model also achieves the highest F1-score, indicating an optimal balance between precision and recall.

## 1. Introduction

Vehicle detection based on machine vision has been widely applied in the field of intelligent transportation. Due to limited coverage of urban road areas by fixed surveillance cameras, UAV-based inspection provides a flexible and efficient alternative for detecting vehicles in target regions to support the development of intelligent transportation systems (ITS) and ensure smooth and efficient urban traffic flow. In ITS, accurate vehicle detection is of vital importance [1,2]. However, in real-world vehicle detection tasks, challenges such as complex image backgrounds, large variations in vehicle scale, and geometric distortion caused by camera angles significantly degrade detection accuracy. Though once dominant, traditional object detection techniques are increasingly unable to cope with the growing demands of modern visual perception tasks due to issues such as high computational overhead and limited robustness under complex real-world conditions [3]. As a result, researchers have gradually shifted their focus towards deep learning-based approaches, which have demonstrated significant potential and adaptability in the domain of vehicle detection. With the continuous advancement of deep neural networks, object detection methods powered by deep learning have seen widespread application. Current methods can be broadly divided into the categories of one-stage and two-stage frameworks [4]. Representative two-stage models include algorithms such as Faster R-CNN [5] and R-FCN [6], whereas popular one-stage models feature designs such as SSD [7], RetinaNet [8], and the increasingly influential YOLO series. In terms of two-stage methods, various researchers have proposed enhancements to further boost their performance. For instance, Peng et al. [9] introduced a series of improvements to the classical Faster R-CNN by refining aspects such as training strategies, convolutional module configurations, and the selection of anchor boxes. These modifications contributed to notable gains in both classification accuracy and localization precision. Song et al. [10] took a different route by redesigning the feature extraction pipeline, specifically by substituting the original backbone network and adjusting the loss function structure. This approach ultimately led to enhanced detection outcomes, especially for vehicle targets. Hu et al. [11] made notable progress by integrating deformable convolutional networks into the training pipeline of R-FCN, enhancing the model’s flexibility and responsiveness when dealing with objects of varying shapes, scales, and spatial orientations. This enhancement significantly improved the detector’s robustness in complex scenarios. As a general rule, two-stage object detection models follow a sequential process. They first generate a set of region proposals or candidate areas of interest, which are then passed through additional network layers for object classification and bounding box regression [12]. Although this cascaded design contributes to accuracy, it inevitably introduces drawbacks such as elevated computational burden, increased training complexity, and slower inference speed. These factors collectively make two-stage methods less ideal for applications such as on-road vehicle detection that require real-time responsiveness. In contrast, one-stage object detectors streamline the entire detection process by eliminating the region proposal step and directly performing classification and localization in a single unified stage. This architectural simplification enables significantly faster inference, making these methods more suitable for real-time environments. For example, Song et al. [13] enhanced the traditional SSD framework by incorporating a learnable low-pass filtering mechanism with anti-aliasing properties, which helps to mitigate distortion of the feature maps. Additionally, they retained high-resolution feature layers with the aim of enhancing the detection accuracy for small-sized objects, leading to greater overall precision, especially in challenging detection tasks involving vehicles of varying sizes. Zhang et al. [14] introduced octagonal convolution and a weighted pyramid structure into RetinaNet to improve detail representation in feature maps. Although SSD and RetinaNet show better real-time performance compared to two-stage models, SSD relies on shallow features for small object detection. These features may lack sufficient semantic information, often resulting in missed detections or inaccurate localization. While RetinaNet improves dense small-object detection by incorporating Feature Pyramid Networks (FPNs) and focal loss, it suffers from high computational costs that limit its real-time application in vehicle detection. The YOLO algorithm was first proposed by Redmon et al. in 2016 [15] and has been widely adopted for various object detection tasks thanks to its high accuracy and fast inference speed. The YOLO series is a high-performance and constantly-evolving detection framework, making it highly appropriate for vehicle detection tasks. To mitigate missed vehicle detections, Jiang et al. [16] combined K-means clustering with YOLOv3 to improve the accuracy of bounding box size estimation, resulting in enhanced detection performance for small vehicles. Mustafa et al. [17] integrated the CBAM attention mechanism into YOLOv4, allowing the model to more effectively concentrate on the crucial features of vehicles. Using YOLOv5s as the baseline, Fan et al. [18] integrated weighted box fusion to address inaccurate prediction counts and introduced attention mechanisms to improve detection speed and accuracy. Zhang et al. [19] enhanced the YOLOX model by adding the NAM attention mechanism to suppress redundant non-salient features, and additionally introduced a small-object detection head to improve sensitivity to small vehicles. Shi et al. [20] optimized the C2f and SPPF modules in YOLOv8, improving feature extraction for vehicles of different sizes and reducing background interference. Zhao et al. [2] proposed the YOLO-BOS algorithm, introducing a novel BRSA attention mechanism and integrating ODConv to improve detection accuracy for dense vehicle targets by modifying the loss function. Bai et al. [3] developed the SFFEF-YOLO algorithm, which optimizes information extraction through a fine-grained feature extraction module and enhances small object detection accuracy in UAV perspectives by integrating multi-scale feature fusion. Fonod et al. [21] optimized the YOLOv8s algorithm by increasing the input resolution to 1080 p and adjusting the IoU threshold to enhance vehicle detection accuracy from a UAV bird’s-eye view. However, these methods still fail to address the significant target scale variations and dense vehicle distributions in practical urban road vehicle detection tasks using UAVs as a result of varying flight altitudes and camera angles, resulting in insufficient detection accuracy under low-altitude UAV perspectives.

Vehicle target detection in low-altitude UAV perspectives faces three major technical challenges: first, the dynamic flight of UAVs causes significant variations in target scales due to altitude changes, making it difficult for conventional convolutional networks with fixed receptive fields to effectively capture multi-scale features and leading to insufficient representation of small vehicle targets in shallow layers; second, vehicle distributions in complex urban scenes exhibit regional patterns, while implicit encoding of coordinate information fails to effectively model the absolute and relative spatial positions of targets, reducing the model’s spatial sensitivity and impairing detection accuracy; third, aerial images often contain vehicle targets with small pixel proportions, blurred contours, or deformations as well as targets partially occluded by adjacent vehicles due to dense parking. The conventional YOLO loss function has limitations in gradient optimization for such challenging samples, resulting in inadequate convergence. To address these challenges, this paper proposes an improved algorithm based on the YOLOv11s model that effectively reduces false positives and missed detections while enhancing detection accuracy. The main contributions of this work are as follows:

1. A novel collaborative optimization strategy is proposed. This strategy enhances the original C3K2 module and replaces its Bottleneck component with a Dilation-Wise Residual Module while integrating an SE attention mechanism to improve sensitivity to vehicle features. Furthermore, the loss function is replaced with the Focaler-WIoU loss, enabling collaborative dual-path enhancement by combining attention-augmented feature extraction with robust learning from low-quality samples. This approach improves both detection accuracy and model robustness.
2. The convolutional layers in the Neck component are replaced with CoordConv (coordinate convolution) to strengthen the model’s spatial awareness. By incorporating explicit coordinate learning, the model achieves more precise localization of vehicle targets.
3. A small object detection head is introduced to enhance the model’s capability in learning features from vehicles of various scales, significantly improving detection accuracy for small vehicles.

In addition, a custom urban road vehicle detection dataset is constructed. This dataset contains a large number of labeled vehicle instances and is characterized by high-resolution (up to 4 K) UAV-captured images with smoothly varying altitudes and stabilized camera gimbal angles. The dataset is designed to be efficient in terms of training resource consumption while supporting improved detection accuracy when used in conjunction with the proposed algorithm.

## 2. Theoretical Method

In this research, the YOLOv11s model is adopted as the foundational framework for subsequent enhancement. YOLOv11, officially released by Ultralytics on 30 September 2024, is an advanced iteration of the YOLO series featuring notable structural improvements over earlier versions such as YOLOv5 and YOLOv8. These upgrades contribute to its superior performance in object detection tasks. Among its different versions, YOLOv11s was selected because it strikes a good balance between detection precision and computational efficiency. This characteristic makes it a fitting choice for situations with limited resources.

**Figure 1:**
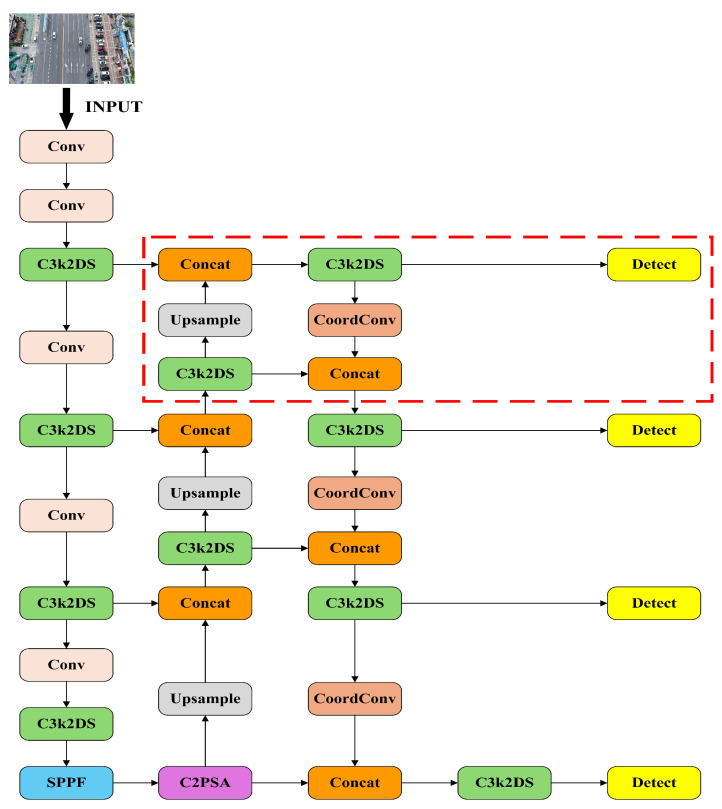
DSCW-YOLO model.

To address the difficulty of small object detection in UAV-acquired images, a collaborative optimization strategy is introduced. This strategy integrates an improved C3K2 module and substitutes the Focaler-WIoU loss for the conventional CIoU loss, aiming to enhance the model’s precision in identifying small-scale vehicle targets. Furthermore, coordinate convolution (CoordConv) is incorporated into the Neck component of the architecture to strengthen the network’s spatial representation capabilities. In addition, a supplementary detection head is added specifically for small object recognition, further reinforcing the model’s multi-scale detection capacity. Collectively, these architectural modifications contribute to a reduction in both false positives and missed detections while simultaneously boosting overall detection performance. Figure 1 depicts the structural layout of the enhanced YOLOv11s model, in which the area enclosed by the red rectangle corresponds to the supplementary detection head.

### 2.1. Collaborative Optimization Strategy of the C3K2DS Module and Focaler-WIoU Loss Function

**Figure 2:**
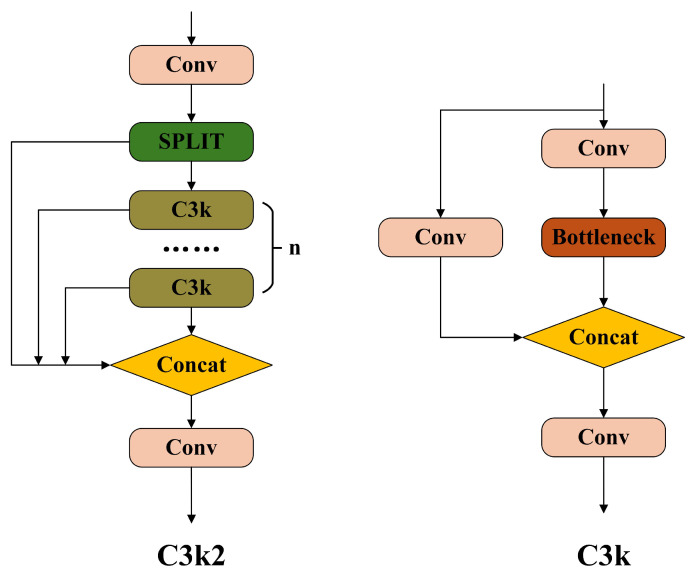
C3K2 module and C3K module.

In the YOLOv11 architecture, the C3K2 module includes a configurable C3K parameter that determines whether to activate the module. When disabled, the Bottleneck module is used in its place. The C3K module supports adjustment of convolutional kernel sizes to enhance the capture of features at various scales. Its architectural details are visualized in Figure 2. Importantly, increasing the kernel size of the C3K module introduces a large number of parameters, which may cause deep features to lose critical details from shallower levels. On the other hand, using a smaller 1 × 1 convolutional kernel fails to expand the receptive field, making it more difficult to capture spatially adjacent features.

In the context of vehicle detection from UAV perspectives, the use of a fixed 3 × 3 convolution kernel within the C3K module results in a limited receptive field that impairs global context modeling capabilities. This constraint hampers the model’s ability to adapt to targets of diverse scales and intricate backgrounds. As a result, it proves inadequate for reliable vehicle detection in UAV-related scenarios.

**Figure 3:**
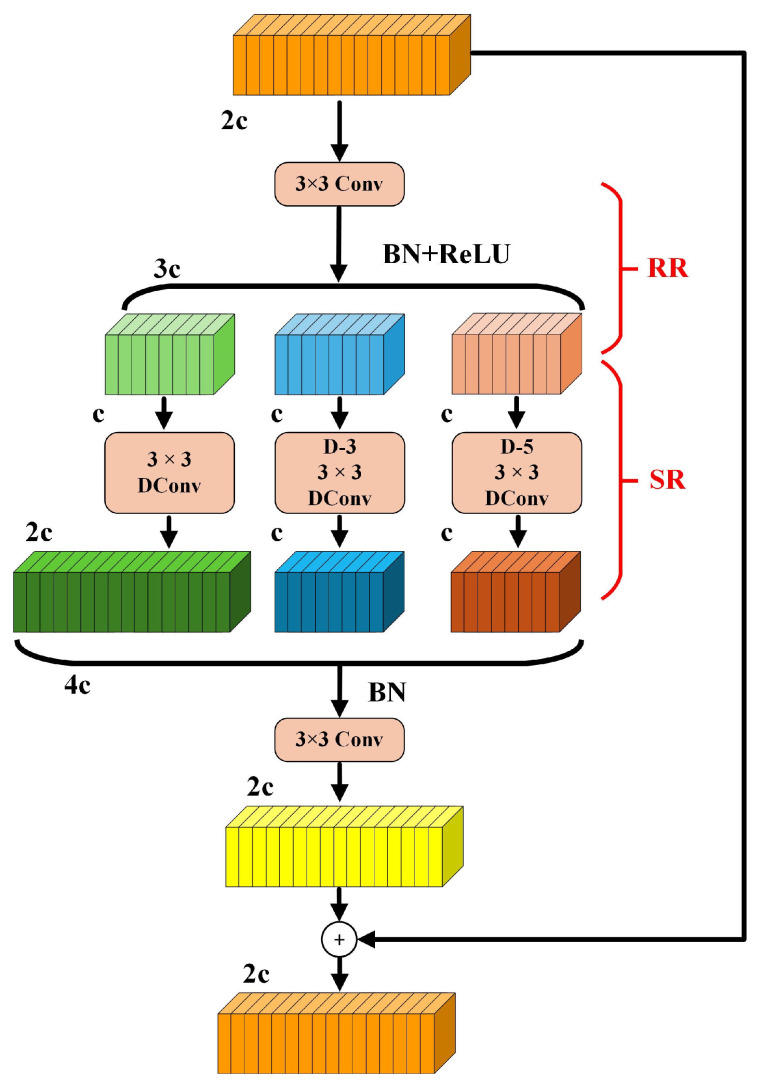
The DWR module.

To address the aforementioned limitations, this paper introduces the Dilation-Wise Residual (DWR) structure into the C3K2 module. The proposed structure enhances multi-scale receptive fields and improves gradient propagation in deep networks. The architecture of the DWR module is illustrated in Figure 3 [22].

The DWR module is designed with a two-stage architecture that integrates both regional and semantic residual enhancements. Initially, the input feature map undergoes a 3 × 3 convolution operation coupled with a nonlinear activation function, which serves to reduce feature complexity while maintaining essential spatial structure. Subsequently, multiple parallel dilated convolution layers with dilation rates set to d = 1, 3, and 5 are applied to perform morphological filtering across regions of different sizes. This allows the network to capture contextual information from both local and mid-range receptive fields, thereby improving its responsiveness to objects across a variety of scales. To further support stable gradient flow and ensure effective end-to-end learning, residual pathways are incorporated in order to mitigate issues such as gradient vanishing in deeper network layers.

**Figure 4:**
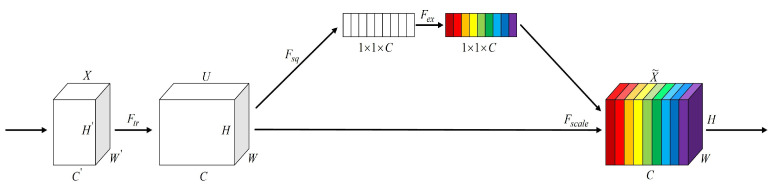
Architecture of the SE attention.

In aerial images captured by UAVs over urban and suburban areas, different forms of background interference such as intricate building textures, shadow patterns, and inconsistent illumination can significantly disrupt the recognition of vehicle targets. Such interference often results in mis- or missed detections. To mitigate the adverse impact of such background elements on detection performance, this study incorporates the SE attention mechanism [23] into the C3K2 module. A visual representation of the SE attention structure is provided in Figure 4.

Through the Squeeze phase, the SE mechanism applies global average pooling to summarize spatial information along each channel, contributing to the suppression of unwanted high-frequency signals. Subsequently, fully connected layers (Excitation phase) are used to generate channel-wise attention weights, which emphasize detailed vehicle features while reducing the response to background noise.

The improved model proposed in this paper is based on YOLOv11s. In the backbone network, the last two C3K2 modules and the C3K2 module preceding the large-object detection head have their C3K configuration enabled, while the remaining C3K2 layers use the default Bottleneck structure.

In this work, the Bottleneck components in all C3K2 modules of the original YOLOv11 architecture are replaced with the DWR structure. For those C3K2 modules that have C3K enabled, the Bottleneck units within the C3K submodules are also replaced by DWR. Furthermore, the SE attention mechanism is integrated into the C3K2 modules, forming the improved C3K2DS module.

**Figure 5:**
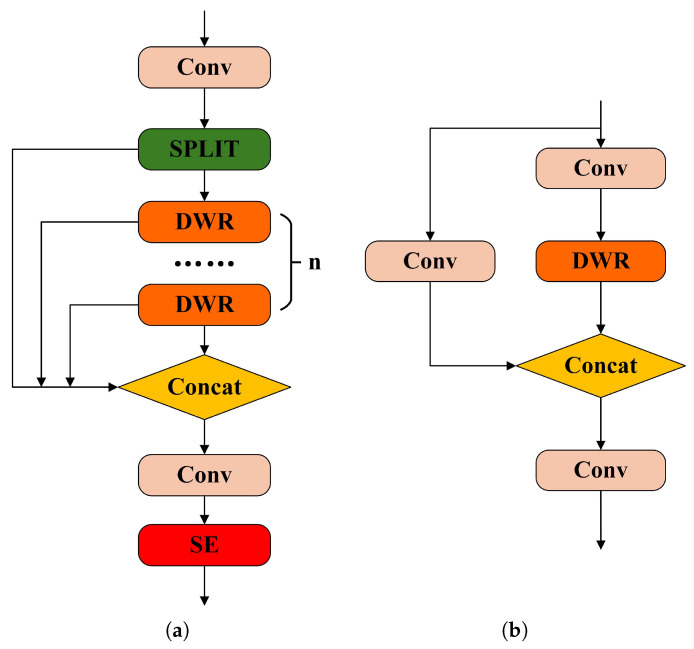
(**a**) C3K2DS module and (**b**) C3K with DWR module.

Figure 5 presents the configurations of the updated modules, with (a) detailing the C3K2DS architecture and (b) showcasing the C3K variant incorporating the DWR structure.

The proposed C3K2DS module incorporates three parallel dilated convolutional branches, each utilizing a distinct dilation rate; the branch with d = 1 is designed to emphasize local feature extraction, effectively preserving fine-grained information critical for identifying small vehicle targets. In contrast, the branches with dilation rates of d = 3 and d = 5 serve to broaden the receptive field. This enables the network to capture the overall shapes and structural outlines of vehicles, strengthening its global feature representation capability. To further refine feature focus, the SE attention mechanism is embedded into the module, which aids in suppressing irrelevant background interference. This enhancement allows the network to concentrate more precisely on vehicle regions to alleviate the impact of uneven lighting and background complexity, ultimately contributing to a reduction in missed detections.

**Figure 6:**
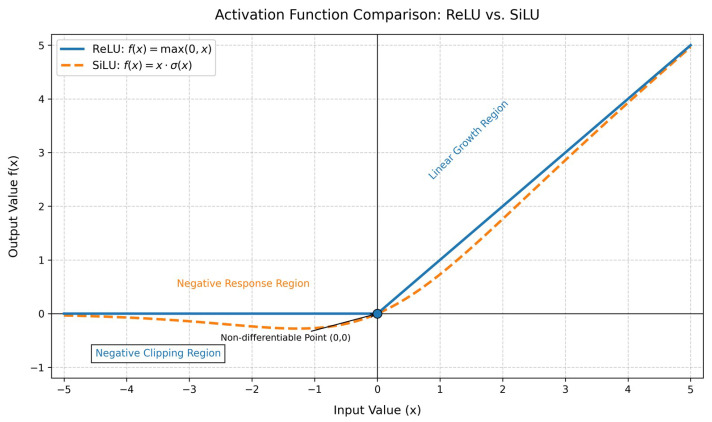
Comparative analysis of the ReLU and SiLU activation functions.

The original DWR structure uses the ReLU activation function following the first 3 × 3 convolution. However, characteristics of ReLU such as hard thresholding, abrupt gradient transitions, and static activation thresholds make it poorly suited to the challenges posed by complex aerial scenes in UAV-based vehicle detection tasks. In contrast, the SiLU (Sigmoid Linear Unit) activation function offers smoother activation, retains negative values, and provides dynamic responses, making it better suited for UAV imagery characterized by non-uniform lighting and large variations in object scale. A comparison between the ReLU and SiLU activation functions is shown in Figure 6. To further improve module performance, in this study we replace the ReLU activation function in the DWR module’s first 3 × 3 convolution layer with the SiLU function.

In practical applications, UAV-captured images are affected by the combined influence of camera angle, flight altitude, and lighting conditions. Due to small gimbal angles and wide shooting ranges, certain vehicle targets may occupy only a small pixel proportion in the image, making them difficult to detect. Additionally, some vehicle targets may exhibit geometric distortions or deformations, further complicating target identification. Moreover, vehicles parked side by side may result in overlapping bounding boxes due to partial occlusion by adjacent vehicles, which can interfere with the model’s ability to accurately learn target features. These challenging low-quality samples increase the difficulty of feature learning for the model.

To tackle this challenge, the proposed model adopts an improved loss computation scheme by introducing the Focaler-WIoU loss in place of the conventional loss function. This change diminishes the detrimental effects of hard samples on detection performance.

In contrast to conventional IoU-based loss functions, Focaler-IoU [24] introduces a piecewise linear interval mapping strategy to reformulate the IoU loss, resulting in improved performance in bounding box regression. Computation of the Focaler-IoU loss is defined in Equations (1) and (2). The parameters *d* and *u* in the equations are tunable within the range [*d*, *u*] ∈ [0, 1].

(1) $$IoU_{\mathrm{focaler}}=\left\{\begin{array}{cc} 0, & IoU<d\\ \frac{IoU-d}{u-d}, & d\ll IoU\ll u\\ 1, & IoU>u \end{array}\right.$$

(2) $$L_{\mathrm{Focaler}\text{-}\mathrm{IoU}}=1-IoU^{\mathrm{focaler}}$$

For the dataset adopted in this study, the Focaler-IoU loss improves the influence of low-quality samples within the loss computation, enhancing the model’s capacity to learn from extremely small vehicle targets and heavily degraded instances. Meanwhile, WIoU [25] introduces a dynamic scaling mechanism that adjusts the loss weight in accordance with the current IoU value. This adaptive strategy increases the penalty for hard samples exhibiting low IoU, effectively guiding the model to pay greater attention to challenging and low-quality targets. In this work, the final loss function is formulated by integrating the advantages of both Focaler-IoU and WIoU, with the mathematical definitions provided in Equations (3)–(5).

(3) $$L_{\mathrm{Focaler}\text{-}\mathrm{IoU}}=1-\left[\mathrm{clip}\left(\frac{IoU-d}{u-d},0,1\right)\times R\right]$$

(4) $$R=\frac{\beta }{\delta \cdot \gamma^{\beta -\delta }}$$

(5) $$\beta =\frac{IoU}{\mu_{IoU}}$$

In the above equations, *R* represents the scaling factor of the loss function, the parameters γ and δ are tunable hyperparameters, and the variable $\mu_{IoU}$ denotes the global average IoU, which is used to adaptively adjust the scaling factor. The $\mu_{IoU}$ is obtained using Equation (6), where the α parameter is a momentum term.

(6) $$\mu_{IoU}^{\left(t\right)}=\left(1-\alpha \right)\cdot \mu_{IoU}^{\left(t-1\right)}+\alpha \cdot \frac{1}{N}\sum_{i=1}^{N}IoU_{i}^{\left(t\right)}$$

First, the Focaler-IoU adjusts the IoU distribution to reshape the loss landscape; then, the scaling factor from the WIoU further refines the loss weight based on the ratio between the current sample’s IoU and the global average IoU. This dual-stage strategy enables more flexible learning, allowing the model to better adapt to the challenges posed by complex urban backgrounds.

In UAV-based vehicle detection tasks within urban environments, the YOLOv11s model suffers from insufficient ability to extract fine-grained features of small objects and limited robustness to challenging low-quality samples. These deficiencies often result in false detections and missed targets. To address this issue, in this study we propose a collaborative optimization strategy that combines the C3K2DS module with the Focaler-WIoU loss function.

Specifically, the C3K2DS module enhances the model’s ability to represent vehicle features by integrating cross-level feature fusion and channel-wise attention mechanisms, thereby improving the representation of fine details. Meanwhile, Focaler-WIoU dynamically adjusts the loss weight based on classification difficulty and localization quality, strengthening the model’s ability to learn hard samples.

This strategy effectively reduces false positives and missed detections, resulting in significantly enhanced vehicle detection accuracy in UAV imagery.

### 2.2. Coordinate Convolution (CoordConv)

Traditional convolution operations extract features using a sliding window mechanism based on local receptive fields, which inherently ignores both the absolute and relative positional information of the pixels. This spatial invariance poses limitations when detecting small or occluded vehicle targets in UAV-captured imagery. In the YOLOv11s model, the Neck component employs conventional convolution layers that lack explicit spatial awareness. This may result in insufficient feature extraction for vehicle targets, potentially affecting detection accuracy.

**Figure 7:**
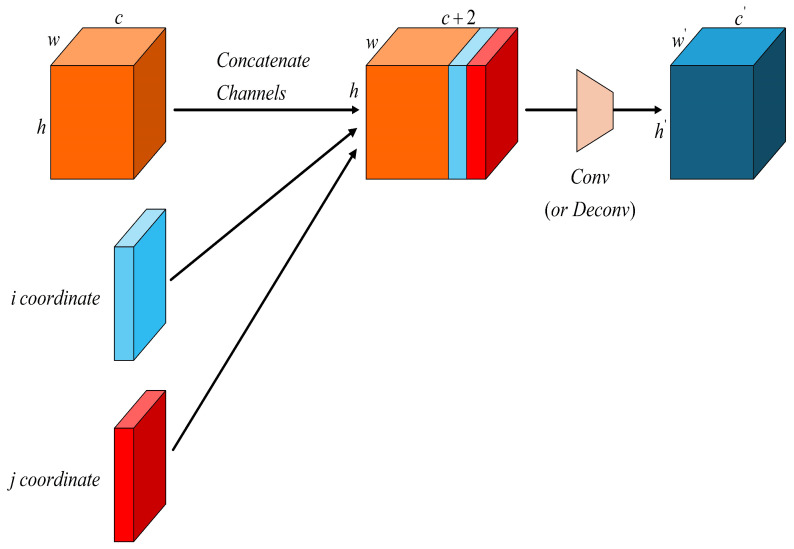
Architecture of the CoordConv module.

In contrast to standard convolution, Coordinate Convolution (CoordConv) [26] introduce additional x-coordinate and y-coordinate channels appended to the input feature map. The structure of the CoordConv operation is illustrated in Figure 7. As shown in the figure, during the forward pass, the input feature map is augmented with coordinate values i and j, which are stacked alongside the original feature map and then processed by convolutional filters. This explicit injection of positional information enables the model to better capture spatial relationships, improving localization precision, especially for small and irregularly distributed vehicle targets in UAV scenes.

Replacing the traditional convolution in the Neck part of the YOLOv11s model with Coordinate Convolution (CoordConv) allows the network to explicitly perceive spatial information by introducing coordinate channels. This enhances the model’s ability to learn both the absolute and relative positional information of vehicle targets. With improved spatial awareness, the model can more accurately localize small or partially occluded vehicles, effectively reducing missed detections.

Moreover, the spatial distribution of vehicles in urban environments often follows predictable patterns; for example, vehicles tend to cluster in areas such as parking lots or roadways, while appearing less frequently in green spaces or rural farmland. By incorporating CoordConv, the model is able to learn the correlation between spatial priors and target distributions, improving its efficiency in capturing context-dependent spatial patterns and enhancing its overall detection performance.

### 2.3. Small Object Detection Head

Variations in UAV flight altitude and camera gimbal angle directly affect the apparent scale of vehicle targets in captured images. As the YOLOv11s model has only three detection heads by default, these scale changes make it challenging to effectively detect vehicle targets across all size ranges. The inherent size differences among vehicles (e.g., between compact cars and large buses) further exacerbate this challenge. The aerial perspective of UAV imagery tends to amplify such scale variations, making it difficult for YOLOv11s to maintain consistent detection accuracy for both small- and large-scale targets.

In this paper, an extra P2 detection head is introduced into the YOLOv11s architecture to specifically improve small-object recognition, increasing the number of detection layers from three to four. This architectural adjustment significantly extends the model’s scale adaptability, especially for small target detection. By capturing finer-grained features at earlier stages in the feature pyramid, the proposed modification effectively mitigates the accuracy degradation caused by scale variation resulting from changes in UAV altitude and viewing angle.

## 3. Experiments and Analysis

### 3.1. Experimental Setup

The experimental setup employed in this research is presented in Table 1.

The learning rate was maintained at 0.01 throughout, with both the initial (lr0) and final (lrf) values set identically.

### 3.2. Dataset

This study presents a custom dataset named DJCAR that is specifically designed for vehicle detection from UAV perspectives in urban environments. In the field of UAV-based vehicle detection, most existing studies rely on publicly available datasets such as VisDrone2019 [27,28], UVADT [29], and CARPK [30], which either partially or fully focus on vehicle detection from aerial views.

A detailed comparison between the proposed DJCAR dataset and the above three datasets is presented in Table 2.

Although all of the above datasets are designed for UAV-based object detection tasks and contain a large number of vehicle targets, VisDrone2019 and UVADT have extremely large data volumes, which imposes high demands on training resources. Additionally, both datasets suffer from low image resolution, typically below 2 K. In the case of VisDrone2019, the dataset includes a wide variety of object categories beyond vehicles, which reduces its specificity for vehicle detection. On the other hand, UVADT features a wide range of UAV flight altitudes, making it less focused on consistent urban road scenarios.

These characteristics limit the applicability of VisDrone2019 and UVADT to urban road vehicle detection tasks.

By contrast, the CARPK dataset is specifically focused on vehicle detection in parking lots, resulting in overly uniform backgrounds. Furthermore, its UAV images are captured at a fixed altitude of approximately 40 m, which does not simulate the dynamic altitude variations encountered in real-world UAV flights. Consequently, CARPK lacks generalization for more complex UAV-based vehicle detection scenarios.

**Figure 8:**
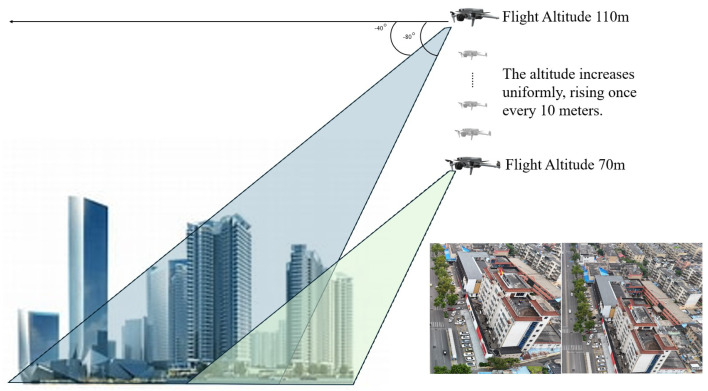
Image examples from the DJCAR dataset.

The proposed DJCAR dataset was collected using a DJI drone, specifically the DJI Air 3 model. The data were captured in urban road environments. The data collection procedure is illustrated in Figure 8. The flight altitude of the drone was increased progressively from 70 m to 110 m, with an increment of 10 m at each step. Simultaneously, the camera gimbal angle varied from −40° to −80° in steps of 10°.

During image acquisition, the drone first ascended to a height of 70 m. At this altitude, the camera gimbal started at an angle of −40° to capture images. After completing the image capture process at this angle, the gimbal rotated to −50°, then to −60°, and continued until −80°, all while maintaining a constant flight altitude. After all gimbal angles were covered at 70 m, the drone ascended to 80 m and repeated the same gimbal rotation and image capture process. This procedure continued until image collection was completed at all planned altitudes and gimbal angles.

During collection of the DJCAR dataset, the UAV’s flight altitude was varied uniformly at 10-meter intervals, covering a range from 70 m to 110 m. All images were stored in separate folders according to the corresponding flight altitude, facilitating research on vehicle detection tasks at specific altitudes. In contrast, other datasets do not include such altitude annotations, making it difficult to obtain flight altitude information.

**Figure 9:**
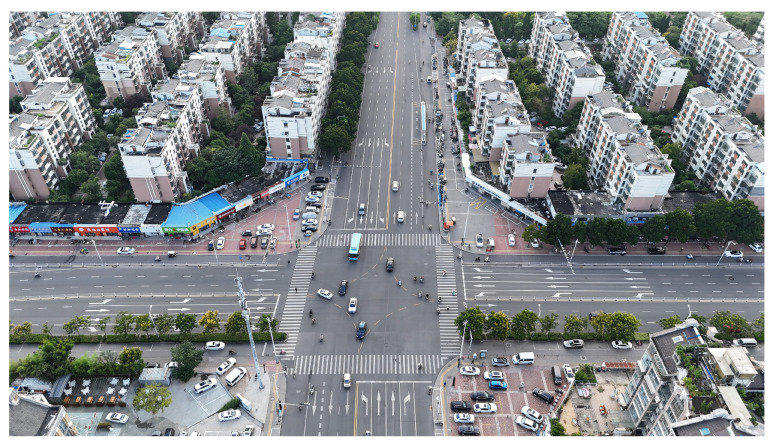
Examples of images from the DJCAR dataset.

Representative sample images from the dataset are shown in Figure 9.

The dataset contains a total of 949 images, with 26,074 annotated vehicle instances. Each image has a resolution of 4032 × 2268, achieving 4 K-level clarity. The DJCAR dataset features rich and diverse backgrounds, high image quality, and a sufficient number of labeled vehicle samples to meet the requirements of vehicle detection tasks. During the vehicle target annotation phase, we adopted the following labeling method. The annotated vehicle target was positioned at the center of the bounding box while ensuring that the bounding box also included a portion of the surrounding background. This approach enhances the model’s ability to learn vehicle features in different road environments. In the DJCAR dataset, targets with complete and clearly defined contours are classified as simple samples. Targets with small pixel proportions, geometric distortions or deformations, or partial occlusions caused by adjacent parked vehicles resulting in overlapping bounding boxes are classified as challenging low-quality samples. Finally, extremely low-quality targets such as those with excessive occlusion or severe blurring are excluded from annotation to maintain dataset quality, as are targets that are indiscernible to the human eye as vehicles. This quality control approach ensures that all annotated targets, including both simple and challenging samples, are high-quality targets with valuable learning potential.

Compared with the three commonly used datasets listed in Table 1, the DJCAR dataset requires significantly fewer training resources while offering greater task relevance for UAV-based urban vehicle detection.

### 3.3. Comparative Experiments

In this study, the performance of different algorithms is evaluated using four commonly adopted metrics: Precision (*P*), Recall (*R*), *F*1-score, and Mean Average Precision (*mAP*). The specific calculation formulas are as shown below.

(7) $$P=\frac{TP}{TP+FP}\times 100\%$$

(8) $$R=\frac{TP}{TP+FN}\times 100\%$$

(9) $$F1=\frac{2\times (P\times R)}{P+R}$$

(10) $$AP=\int_{0}^{1}P\left(r\right)\;dr$$

(11) $$mAP=\frac{\sum_{i=1}^{C}AP_{i}}{C}$$

In the presented formulas, *TP* (true positives) corresponds to correctly predicted positive instances, *FP* (false positives) indicates incorrect positive predictions made on negative samples, *FN* (false negatives) refers to actual positive cases that the model failed to detect, *AP* quantifies the precision averaged across recall levels for a single class, and *mAP* provides a global performance metric by averaging *AP* scores over all classes.

In our algorithm comparison experiments, in addition to commonly used YOLO series models, we also include the latest version, YOLOv12 [31]. Furthermore, to ensure a comprehensive evaluation, the comparison also involves two of the latest detection frameworks, RT-DETR [32] and DEIM-Dfine [33]. According to the results summarized in Table 3, the method proposed in this study delivers the highest performance across all assessed indicators.

Compared with the original YOLOv11s model, the proposed algorithm achieves an improvement of 1.9% in precision, 6.0% in recall, 4.2% in mAP@0.5, and 3.3% in mAP@0.5:0.95. Moreover, the F1-score reaches the highest value among all models, indicating that the proposed algorithm not only improves detection accuracy but also maintains a better balance between precision and recall.

To evaluate the real-time performance of the improved model, a runtime test was conducted on 4 K-resolution images. The complete processing pipeline encompassing preprocessing, model inference, and post-processing achieved a speed of 73.87 frames per second (FPS), satisfying the real-time performance demands of UAV-based vehicle detection tasks.

Additionally, a comparative analysis was carried out among the top-performing models: YOLOv8s, YOLOv11s, and our proposed approach. The detailed results are presented in Table 4.

Based on the statistical analysis, the detection results for Image a, Image b, and Image c in Table 4 are summarized in Table 5.

In the results for Image a, both YOLOv8s and YOLOv11s exhibit a significant number of missed detections for the densely distributed vehicles in the upper-right area of the image, whereas the proposed model achieves near-complete detection with minimal omissions.

In the results for Image b, only the proposed model successfully avoids both false positives and missed detections; in contrast, YOLOv8s and YOLOv11s both mistakenly detect a trash bin in the center of the image as a vehicle, and both also misclassify a three-wheeled vehicle near the building on the left as a standard car. This demonstrates that the proposed model can effectively handle complex backgrounds and small-scale vehicle targets, resulting in fewer false and missed detections.

In the results for image c, both YOLOv8s and YOLOv11s mistakenly detect the window in the upper left corner as a vehicle, and a vehicle in the upper middle area is missed as well. Images d and e are both captured images with dynamic blur added through code. In image d, the vehicles in the left-side parking area are highly blurred, leading to a certain amount of missed detections across all three models. However, the proposed model identifies two more vehicle targets in densely populated and blurred areas compared to the other two models. In the results for image e, the proposed model reduces false detections by two while maintaining no missed detections compared to YOLOv8s and YOLOv11s, demonstrating its robustness in handling blurred images by effectively mitigating false and missed detections.

### 3.4. Ablation Study

To evaluate the impact of each proposed improvement on the YOLOv11s model, a series of ablation experiments were conducted. For all tables in this subsection, a value of “✓” indicates that the corresponding component was included in the ablation configuration. First, an ablation study was performed on the collaborative optimization strategy involving the C3K2DS module and the Focaler-WIoU loss function. In these experiments, A denotes the C3K2DS module and B denotes the Focaler-WIoU loss. Table 6 presents the experimental results.

As shown in Table 6, when the C3K2DS module is added individually, precision (P) and recall (R) increase by 0.6% and 0.4%, respectively, with an improvement of 0.5 in the F1-score. This indicates that the C3K2DS module enhances the model’s ability to extract features from small vehicle targets, achieves a good balance of precision and recall, and improves the model’s overall robustness. However, using the C3K2DS module alone results in a 0.3% drop in mAP@0.5:0.95, suggesting that it may slightly compromise localization precision under stricter IoU thresholds.

When the Focaler-WIoU loss is applied alone, precision and recall improve by 0.4% and 0.9%, respectively, with the F1-score increasing by 0.7. Additionally, both mAP@0.5 and mAP@0.5:0.95 show slight improvements. These results demonstrate that the Focaler-WIoU loss contributes positively to improving the model’s learning on hard or low-quality samples.

When the two components are used jointly, both precision and recall exhibit a significant increase of 2.0%, the F1-score improves by 1.6, and the mAP@0.5 increases by 1.3%,which outperforms the individual contributions of each module. Although the mAP@0.5:0.95 of the combined strategy is 0.4% lower than when using the Focaler-WIoU loss alone, it is important to note that false detections and missed detections can have serious consequences in urban vehicle detection tasks; therefore, minimizing false positives and false negatives is of higher priority than achieving marginal gains in high-precision localization.

Considering that the drop in mAP@0.5:0.95 is minimal (only 0.4%), while the other four metrics are more critical to the task at hand and all show substantial improvements, it can be concluded that the proposed collaborative optimization strategy offers meaningful enhancements to the detection accuracy of the YOLOv11s model in the context of this study.

To further validate the influence of the proposed collaborative optimization strategy on the precision of detecting vehicle targets from the viewpoints of Unmanned Aerial Vehicles (UAVs), a heatmap comparative experiment was carried out. The comparative results are presented in Table 7.

By comparing the heatmaps of image (a) and image (b) in Table 7, it can be observed that the proposed collaborative optimization strategy demonstrates higher attention to vehicle targets in low-altitude UAV aerial images than YOLOv11s, validating the effectiveness of the proposed strategy.

To further evaluate the contribution of all proposed components, additional ablation experiments were conducted. The collaborative optimization strategy (C3K2DS + Focaler-WIoU) is denoted as A, CoordConv is denoted as B, and the small-object detection head is denoted as C. The experimental results are summarized in Table 8.

According to the experimental results in Table 8, the proposed collaborative optimization strategy alone improves precision (P) and recall (R) by 2.0% over the baseline model, with an increase of 1.6 in F1 score, 1.3% in mAP@0.5, and 0.3% in mAP@0.5:0.95. This indicates that the collaborative optimization strategy effectively reduces false positives and missed detections, resulting in enhanced detection accuracy. However, the 0.3% increase in mAP@0.5:0.95 suggests that the impact on high IoU threshold detection accuracy is limited. Because the primary focus of this study is to minimize false positives and missed detections rather than to ensure precise bounding box localization, the main emphasis should be placed on P, R, and mAP@0.5.

Integrating coordinate convolution (CoordConv) alone improves P by 0.5% and R by 0.8%, indicating its contribution to reducing false positives and missed detections by better capturing spatial priors. Adding the small object detection head to the baseline model results in increases of 1.8% in P, 5.2% in R, and 3.8% in both mAP@0.5 and mAP@0.5:0.95. This demonstrates that the variations in vehicle scale caused by UAV flight altitude and camera angle significantly affect detection accuracy and that the small object detection head can effectively enhance scale sensitivity.

When combining the collaborative optimization strategy and CoordConv, P and R increase by 1.5% and 1.7%, respectively, with a 1.3% improvement in mAP@0.5. However, the improvement in P and R is lower than that achieved by using the collaborative optimization strategy alone. When CoordConv and the small object detection head are combined, P increases by 1.5%, R by 5.1%, and mAP@0.5 by 3.6%. Compared to using only the small object detection head, CoordConv slightly weakens the impact of the small object detection head on mAP@0.5. These two ablation studies suggest that CoordConv fails to fully exploit its potential in enhancing P and R when combined individually with either the collaborative optimization strategy or the small object detection head. This indicates that conflicts may arise between CoordConv and the other two modules when combined in pairs, preventing optimal performance.

When using the collaborative optimization strategy, CoordConv, and the small object detection head together, P and R improve by 1.9% and 6.0%, respectively, F1 score increases by 4.1, mAP@0.5 increases by 4.2%, and mAP@0.5:0.95 increases by 3.3%. Compared to using only the collaborative optimization strategy and the small object detection head, integrating CoordConv further enhances P and R, achieving the highest F1 score. Thus, the combination of all three modules achieves the best overall balance between mAP@0.5, P, R, and the tradeoff between P and R, demonstrating the best performance on the UAV-based vehicle detection task investigated in this study.

## 4. Conclusions

This paper addresses the challenge of vehicle detection from UAV viewpoints in complex urban road environments by presenting a targeted solution that integrates a specially curated dataset and an enhanced detection algorithm based on YOLOv11s that aims to boost detection accuracy.

A novel dataset named DJCAR is constructed for this purpose. It consists of high-resolution 4 K images characterized by diverse and complex background scenes, making it particularly suitable for UAV-based vehicle detection in urban contexts.

On the algorithmic front, this study introduces a collaborative optimization framework that integrates the C3K2DS module with the Focaler-WIoU loss function. This dual approach strengthens the network’s capacity to extract detailed features from small-scale vehicle instances while suppressing background interference and adaptively re-weighting the contribution of low-quality or difficult samples, thereby improving the model’s robustness in challenging scenarios. Furthermore, replacing the conventional convolution in the Neck with coordinate convolution (CoordConv) enhances the model’s ability to learn spatial priors and improves coordinate position perception, thereby increasing vehicle detection accuracy. Additionally, the introduction of a small object detection head enables the model to adapt to multi-scale variations in vehicle targets caused by different UAV flight altitudes and gimbal angles, further improving the detection accuracy of YOLOv11s.

Although the proposed algorithm effectively addresses vehicle detection on urban roads using UAVs, there remains room for further optimization. Future work could focus on vehicle detection under higher IoU threshold requirements by optimizing the algorithm to improve its detection accuracy under stricter IoU conditions. In addition, the proposed DJCAR dataset has room for further expansion. In the future, we plan to incorporate more UAV aerial data under diverse scenes and weather conditions in order to further enhance its generalization capability.

## Figures and Tables

**Table 1:** Experimental configuration.

Item	Specification
Operating System	Windows 10
CPU	Intel(R) Core(TM) i5-14600KF
RAM	32 GB
GPU	NVIDIA GeForce RTX 4080s
CUDA Version	12.1
Python Version	3.9
PyTorch Version	2.2.2
epoch	300
batch-size	16
optimizer	SGD

**Table 2:** Information comparison of the four datasets.

	VisDrone2019	UVADT	CARPK	DJCAR
Vehicle-related Categories	car, bus, van, truck	car, bus, truck	car	car
Flight Altitude	–	10–30 m, 30–70 m, >70 m	about 40 m	70–110 m (Uniform Variation)
Number of Images	8599	80,000	1448	995
Image Resolution	1200 × 1500	1080 × 540	1280 × 720	4032 × 2268
Number of Samples	>540 K	840 K	89,777	26,074

**Table 3:** Comparative experiments with different algorithms.

Algorithms	P/%	R/%	F1-Score	mAP@0.5	mAP@0.5:0.95
RT-DETR	81.0	75.9	78.4	82.1	48.0
DEIM-DFINE	70.4	72.9	71.6	70.4	44.7
YOLOv5s	91.5	82.0	86.5	89.3	56.6
YOLOv7-tiny	87.4	76.5	81.6	85.0	46.3
YOLOv8s	90.7	82.2	86.2	89.3	57.9
YOLOv9s	89.9	81.7	85.6	88.2	56.2
YOLOv10s	87.9	82.3	85.0	89.0	57.3
YOLOv11s	90.1	82.1	85.9	89.4	57.6
YOLOv12s	90.4	81.9	85.9	88.9	56.6
ours	**92.0**	**88.1**	**90.0**	**93.6**	**60.9**

**Table 4:** Comparison of the algorithms’ detection results.

	YOLOv8s	YOLOv11s	Ours
(a)	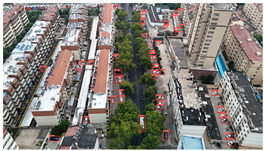	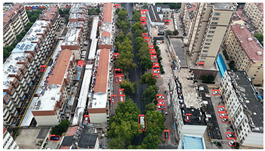	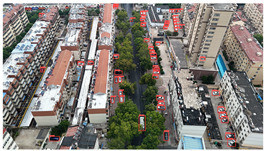
(b)	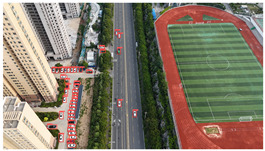	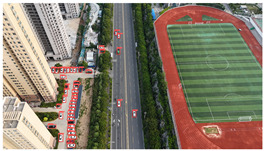	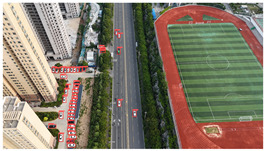
(c)	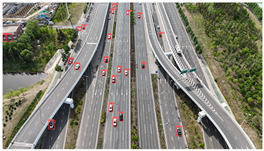	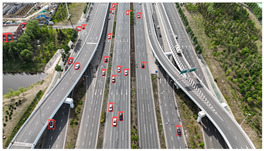	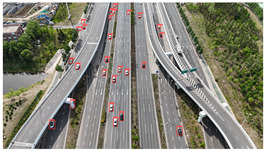
(d)	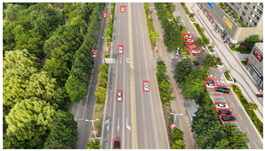	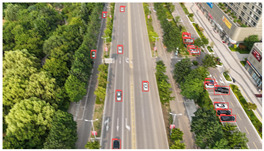	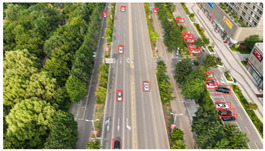
(e)	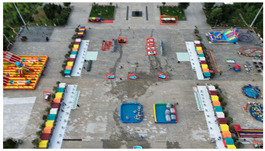	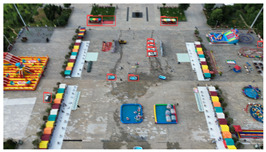	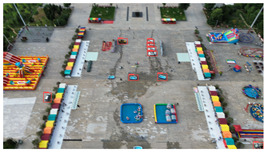

**Table 5:** Detection result statistics of the models on actual images.

Image	Type	YOLOv8s	YOLOv11s	Ours
(a)	Wrong	4	4	**4**
	Missed	17	25	**6**
(b)	Wrong	2	2	**0**
	Missed	4	4	**0**
(c)	Wrong	3	2	**2**
	Missed	9	8	**5**
(d)	Wrong	0	0	**0**
	Missed	10	10	**8**
(e)	Wrong	5	5	**3**
	Missed	0	1	**0**

**Table 6:** Results of the collaborative optimization strategy ablation study.

YOLOv11s	A	B	P/%	R/%	F1-Score	mAP@0.5	mAP@0.5:0.95
✓			90.1	82.1	85.9	89.4	57.6
✓	✓		90.7	82.5	86.4	89.4	57.3
✓		✓	90.5	83.0	86.6	90.1	**58.3**
✓	✓	✓	**92.1**	**84.1**	**87.5**	**90.7**	57.9

**Table 7:** Heatmap visualization comparison.

	Original Image	YOLOv11s	Ours
(a)	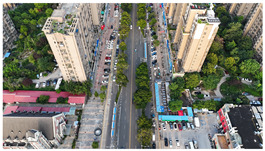	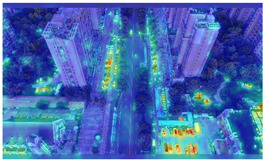	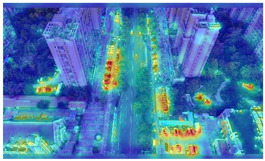
(b)	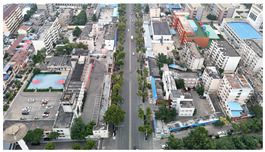	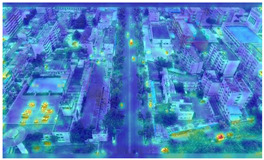	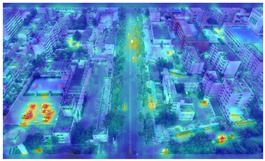

**Table 8:** Ablation experiment results.

YOLOv11s	A	B	C	P/%	R/%	F1-Score	mAP@0.5	mAP@0.5:0.95
✓				90.1	82.1	85.9	89.4	57.6
✓	✓			**92.1**	84.1	87.5	90.7	57.9
✓		✓		90.6	82.9	86.5	90.0	58.3
✓			✓	91.9	87.3	89.5	93.2	61.4
✓	✓	✓		91.6	83.8	87.5	90.7	58.7
✓		✓	✓	91.6	87.2	89.3	93.0	**61.5**
✓	✓		✓	91.7	88.0	89.8	**93.6**	60.9
✓	✓	✓	✓	**92.0**	**88.1**	**90.0**	**93.6**	**60.9**

## Data Availability

The original contributions presented in this study are included in the article. Further inquiries can be directed to the corresponding author.
